# Case of Ulceration of the Duodenum, in Which the Gall-bladder Was Filled with a Colourless Aqueous Fluid, and Contained Numerous Gall-stones

**Published:** 1845-01

**Authors:** C. J. Roberts

**Affiliations:** Physician to the Welsh Charity, &c.


### ROYAL MEDICAL AND CHIRURGICAL SOCIETY.

*Case of Ulceration of the Duodenum, in which the gall-bladder was filled with a colourless aqueous fluid, and contained numerous gall-stones.* By C. J. Roberts, M. D., Physician to the Welsh Charity, &c.—The author only saw this case a short time previous to death. The patient was attacked with violent vomiting on Tuesday evening, which resisted all the means employed to subdue it, and he sank on the following Thursday, at 2 o'clock, P. M. On examination after death, the intestines were very slightly glued together by lymph. The inner membrane of the stomach had many ecchymotic spots on it of a very deep colour. The duodenum was ulcerated through its entire length; the liver small, but healthy; the gall bladder was distended, and at its apex there was a small vesicle more translucent than the other portions of the bladder, looking as if from a rent of the two outer coats, and a protrusion of the inner one. On opening it, more than 4 oz. of a perfectly limpid transparent fluid escaped, but unfortunately none of it could be collected for examination. There were more than a hundred gall stones in the gall bladder, about the size of peas. The kidney and bladder were healthy.

The author remarks that cases where a number of gall stones have been found after death, without their presence causing irritation, or being suspected, are not rare ; but that the total absence of bile, or rather its place being supplied by an aqueous fluid, are not common. He observes, however, that allusions are made to it by some of the older authors, as Fernelius and Haller, and also that mention is made of an altered condition of the biliary secretion, by Andral, and by Drs. Graves and Stokes, as well as by Dr. Thomson, in his Practical Treatise on Diseases of the Liver.

He concludes by observing that the most extraordinary part of the case is, the fact that the man never made any complaint of hepatic derangement during life-time. He was never jaundiced, nor had pain in the right side, notwithstanding one of the calculi was firmly grasped by the duct.

